# Insights on attenuating autophagy cellular and molecular pathways versus methotrexate-induced toxicity via liposomal turmeric therapy

**DOI:** 10.1186/s43141-022-00430-4

**Published:** 2022-10-27

**Authors:** Mai O. Kadry, Naglaa M. Ammar, Heba A. Hassan, Rehab M. Abdel Megeed

**Affiliations:** Therapeutic Chemistry Department, National Research Center, Al Bhoouth Street, Cairo, Egypt

**Keywords:** Methotrexate, FOXO-3, Autophagy, SiRT-1, ERDJ-4

## Abstract

**Background:**

Methotrexate (MX), a competitive inhibitor of dihydrofolate reductase, can inhibit DNA and RNA production and is a powerful anticancer agent widely utilized in clinical practice for treating nonneoplastic maladies, as psoriasis and rheumatoid arthritis; meanwhile, its probable prescription dose and interval of administration are strictly limited due to dose-related organ damage. Former studies verified that kidney, brain, liver, and lung harms are prospective obstacles of methotrexate administration. To understand the machinery of methotrexate-prompt toxicity, various mechanisms were investigated. The former is an autophagy defense mechanism; autophagy is a self-digesting mechanism responsible for the removal of damaged organelles and malformed proteins by lysosome. The contemporary article hypothesized that turmeric or its liposomal analog could defeat autophagy of MX-induced acute toxicity. Methotrexate, in a dose of 1.5 mg/kg, was administered intravenously followed by turmeric and liposomal turmeric treatment in a dose of 5 mg/kg for 30 days in rats.

**Results:**

Increment in autophagy (AUTP) consent by MX administration was attenuated by concurrent treatment via turmeric and liposomal turmeric that was reliable on the alteration in apoptotic markers. The assembly of FOXO-3 in serum post methotrexate administration was suppressed by concurrent treatment via liposomal turmeric. Apoptosis/autophagic marker investigation was evaluated through the gene expression of Bax (BCL2-associated X protein)/Bcl2 (B-cell lymphoma 2)/P53 (tumor protein P53)/SiRT-1 (sirtuin silent mating-type information regulation 2 homolog 1) and FOXO-3 (forkhead box transcription factor-3)/ERDJ-4 (endoplasmic reticulum localized DnaJ homologs)/BNP (brain natriuretic peptide B) signaling. The cell death of all cells was categorized to achieve autophagy. Interestingly, Bax/Bcl2/P53/SiRT-1 signaling pathways were downregulated, contributing to inhibiting the initiation of autophagy. Meanwhile, FOXO-3/BNP/ERDJ-4 reduction-implicated noncanonical autophagy pathways were involved in methotrexate-induced autophagy, whereas this change was suppressed when turmeric was administered in liposomal form.

**Conclusion:**

These outcomes recommended that liposomal turmeric prevents MX-induced acute toxicity through its autophagy, antioxidant, and antiapoptotic properties.

## Background

Autophagy (AUTP) is a catabolic procedure vital for conserving cellular homeostasis and competing cytotoxic insult. Autophagy is documented as “programmed cell survival” contrary to programmed cell death (apoptosis). Exaggerated autophagy was reported in various types of cancers and has been elucidated to both enhance and prevent antitumor drug resistance relying on the duration and nature of the treatment-induced metabolic stress as well as the tumor type. MX, DOX, and cisplatin are widely utilized anticancer drugs [1]. MX, a competitive hinder of dihydrofolate reductase, can obstruct the production of RNA, DNA, proteins, and thymidylate. For this reason, MX is generally utilized in cancer therapy besides nonneoplastic conditions, including psoriasis and rheumatoid arthritis. Previous evidences elucidated that toxicity of the lung, liver, brain, and kidney is the implicit limitations of MX application [2]. To understand MX toxicity, a number of mechanisms have been inspected. One of them is disturbing the antioxidant/inflammatory/apoptotic/autophagosomal defense mechanism, contributing to oxidative stress-induced damage in several organs [2]. MX induced reduction in the antioxidant defense mechanism contributing to exaggerated release of ROS that subsequently enhance mitochondrial malfunction and endoplasmic reticulum stress resulting in hepatorenal toxicity, parenchymal lung injury, and interstitial and alveolar fibrosis [3]. It was evidenced that mitochondrial malfunction and simultaneous ROS production can contribute to autophagic cell death [4]. AUTP is an extremely conserved mechanism that maintains energy resources and gets rid of unnecessary products. It is suitable for reducing stressful conditions as oxidative stress and ER stress. SiRT-1/AKT/mTOR pathway is implied in numerous fibrotic conditions, as liver, cardiac, kidney, and pulmonary fibrosis [5]. Relying on former findings, it is worthy to examine the efficacy of MX on AUTP amelioration via exploring SiRT pathway and its consequence on ERDJ expression that results in toxic influence in addition to inspecting the potential effect of liposomal turmeric in ameliorating these injurious effects. MX can reduce the levels of angiogenic, pro-inflammatory biomarkers, modulate autophagal proteins, and enhance apoptosis by controlling ER stress response. Disturbance in homeostasis between apoptosis and autophagy might be an underlying phenomenon in the progression of resistance to MX [2]. Therefore, it is imperative to combine it with another drug which could decrease its toxicity, increase its efficacy, and target organs. Increasing evidence indicates that autophagy protects various tumor cells from apoptosis induced by chemotherapy drugs, both in vitro and in vivo. Nevertheless, persistent or extensive AUTP also produces cell death. Thus, autophagy often serves as an adapter between cell death and survival [1]. AUTP process eliminates malformed proteins, damaged organelles, and inappropriate long-lived proteins through auto-digestive process via lysosome. AUTP is classified into 3 types relying on the mechanism through which intracellular materials are carried into lysosome for lysis: first is macroautophagy, second is chaperone-mediated autophagy (CMA), and third is microautophagy (1). Macroautophagy implicates the generation of autophagosomes which is subcellular double-membrane-bound structures that contain degradable contents of cytoplasm materials and transfer them into lysosomes for lysis via lysosomal enzymes. Meanwhile, in CMA, proteins flagged via pentapeptide motif (KFERQ) were selectively degraded via direct transfer into lysosome. In microautophagy cytoplasm, material is sequestered via direct engulfment through lysosomal membrane (2). The molecular mechanism of AUTP incorporates various conserved autophagy-related proteins (Atg). Systems produce modified complexes Atg5-Atg12-Atg16 and Atg8-PE as autophagy regulators. AUTP is motivated via diverse physiological and stressful conditions; for instance, hyperthermia and food deprivation in addition to hypoxia that is mediated via factors such as FOXO transcription factors, insulin growth factor-1, m-TOR signaling, and chaperones. Any disturbance in AUTP may contribute to, neuromuscular disorders, myopathies, and cancer (3).

Growing body of evidence validates that AUTP is essential for controlling various intracellular process comprising oxidative stress, differentiation, growth, deficit nutrient, cell death, macromolecule, and organelle turnover (4).

SiRT1, NAD-dependent deacetylase, contributes a part in regulating AUTP. SiRT1 decreases oxidative stress and elevates mitochondrial function and, likewise, was linked to age-related ROS generation, which depends mainly on mitochondrial metabolism. Great relation was observed among SiRT1 activity and induction of AUTP in murine and human embryonic stem cells upon ROS challenge. Hydrogen peroxide prompted apoptosis and AUTP in wild type (WT) and SiRT1/mESCs. Meanwhile, addition of autophagy inhibitor (3-methyladenine) to hydrogen peroxide prompted more apoptosis in WT than in SiRT1/mESCs. Reduced AUTP induction in SiRT1/mESCs was elucidated via reducing the conversion of LC3-I into LC3-II, reduced Beclin-1 expression, and LC3 punctae. Hydrogen peroxide convinced AUTP through loss of mitochondrial membrane eventuality and dislocation of mitochondrial dynamics in SiRT1/mESCs. Exaggerated phosphorylation of P70/85-S6 kinase and ribosomal S6 was observed in SiRT1/ mESCs, revealing that SiRT1 controls the mTOR signaling pathway. This recommends the role of SiRT1 in adjusting AUTP and mitochondria function upon oxidative stress, effects mediated at least in part by the class 3 PI3K/Beclin 1 and mTOR pathways [5].

Turmeric is an active polyphenolic ingredient of *Curcuma longa* with high antioxidant and anti-inflammatory properties that render it an attractive candidate for protection against methotrexate nephrotoxicity. Turmeric has shown renal protective properties against gentamicin- and cisplatin-induced renal toxicities as well as diabetic nephropathy. Turmeric is a SiRT modulator natural product that exhibits poor solubility, and bioavailability utilizing higher bioavailable liposomal loaded preparations of turmeric derivatives may result in elevating SiRT-1-activating action, further authenticating the link between SiRT1, turmeric, and therapeutic effects. Previous studies indicated that the widely held liposomal turmeric (SiRT activator) exerts both direct activating effects and indirect effects via modulation of SiRT1 downstream pathways increasing retention phenomena, targeting organs, increasing bioavilability, and skipping macrophage recognition [6].

The present study therefore aimed to assess the possible therapeutic impact of turmeric and liposomal loaded turmeric and the underlying mechanism(s) responsible for this effect in a rat model of methotrexate-induced toxicity. The mechanism of protection was evaluated by assessing the oxidative stress (malondialdehyde), inflammatory marker (FOXO-3), apoptosis biomarkers (Bax/Bcl2/P53), and autophagy parameters (SiRT-1, BNP, and ERDJ-4).

## Methods

### Chemicals

Methotrexate and turmeric were purchased from Sigma-Aldrich Co. (St. Louis, MO, USA). Kits used were obtained from Randox Company (Antrim, UK). ELISA kit for FOXO-3 determination was obtained from R&D systems (MN, USA), and RT-PCR kits were obtained from the Qiagen Company (USA). All utilized chemicals are of the greatest analytical ranking. Liposomal turmeric was obtained from lipolife company (USA).

### Animals

Male Wistar albino rats, weighing 190–200 g, obtained from the animal house of the National Research Center were utilized in this study. Animals were housed in cages kept at standardized conditions (22 ± 5 °C, 55 ± 5% humidity and 12-h light/dark cycle). They were permitted for free water access in addition to pelleted standard chow diet. All techniques concerning animal maintenance and treatment firmly followed ethical procedures and policies approved by Animal Care (2443042022) and Use Committee of National Research Center.

#### Experimental design

Post 7 days of acclimatization, animals were classified into four groups (ten rats each) and were distributed relying on the following schedule:

1. Group 1: Animals received saline and served as control group.
2. Groups from 2 to 4: Animals were given methotrexate in a dose of 15 mg/kg, and then the following regimen was applied:

- Group 2: Methotrexate-intoxicated animals were left untreated [7].
- Group 3: Methotrexate-intoxicated animals were treated with oral dose of turmeric (15 mg/kg BW) for 1 month [6].
- Group 4: Methotrexate-intoxicated animals were treated with oral dose of liposomal turmeric (5 mg/kg BW) for 1 month [8].

It is worthy to note that the selected dose of methotrexate was previously reported to be the most toxic to most organs [9].

#### Blood sampling

At the end of the experiment, all groups were sacrificed, and blood samples were taken from each animal by puncture of the sublingual vein into sterilized tubes and let it stand for 10 min to clot. Serum was separated by centrifugation at 3000 rpm for 10 min and kept at −80 °C for further determinations of biochemical parameters.

#### Determinations of biochemical parameters

##### Determination of serum malondialdehyde

MDA activity was estimated spectrophotometrically using commercially available kits provided from the Randox Company (UK, Antrim; AS2359) [10].

##### Determination of serum reduced glutathione

GSH activity was estimated spectrophotometrically using commercially available kits provided from the Randox Company (UK, Antrim; AS2359) [11].

##### ELISA determination of serum FOXO-3

Serum FOXO-3 level was measured using ELISA kits (R&D systems MN, USA; no. 112731 and no. BAF583, respectively) according to the manufacturer’s instructions. The assay of this cytokine employs the quantitative sandwich enzyme immunoassay technique. Specific antibody was pre-coated onto the microplate. The standards and samples were pipetted into the wells, and the cytokine was bound by the immobilized antibody. After washing away any unbound substances, an enzyme-linked secondary antibody specific for FOXO-3 was added to the wells [12].

#### Quantitative real-time polymerase chain reaction (qRT-PCR) analysis of serum BNP, P53, Bax, Bcl2, SiRT, and ERDJ mRNA expression

Serum was lysed using QIAzol lysis reagent with a TissueRuptor (Roche). RNA was sequestered via Tripure Isolation Reagent (Roche) relying on the manufacturer’s instructions. To assess the mRNA expression of BNP, P53, Bax, Bcl2, SiRT, and ERDJ, quantitative real-time PCR was accomplished via SYBR green PCR Master Mix (Applied Biosystems, CA, USA). Primer sequences were demonstrated in Table 1 [13].

**Table 1:** Primers sequence designed for RT-PCR gene expression

Primer name	Primer sequence
P53	5′-CAGCGTGA TGATGGTAAGGA-′3 5′-GCGTTGCTCTGATGGTGA-′3
Bcl2	5′-TCTTCAGGCTGGAAGGAGAA-′3 5′-AAGCTGTCACAGAGGGGCTA-′3
Bax	5′-GATCAGCTCGGGCACTTTAG-′3 5′-TGTTTGCTGATGGCAACTTC-′3
GAPDH	5′-CCCTTCATTGACCTCAACTACAATGGT-′3 5′-GAGGGGCCATCCACAGTCTTCTG-′3
SiRT-1	5′-TGGCAAAGGAGCAGATTAGTAGG-3′ 5′-CTGCCACAAGAACTAGAGGATAAGA-3′
ERDJ-4	5′-AGTAGACAAAGGCATCATTTCCAA-3′ 5′-CTGTATGCTGATTGGTAGAGTCAA-3′
BNP	5′-GCAGAAGCTGCTGGAGCTGA-3′ 5′-GATCCGGAAGGCGCTGTCT-3′

### Statistical analysis

Data were represented as mean ± SEM. Statistical analysis was achieved using InStat-3 computer program (GraphPad software Inc., San Diego, CA, USA). One-way analysis of variance (ANOVA) by SPSS 16 program was done followed by post hoc at *p* ≤ 0.05 using Tukey’s test.

## Results

### Modulation of oxidative stress biomarkers

**Fig. 1:**
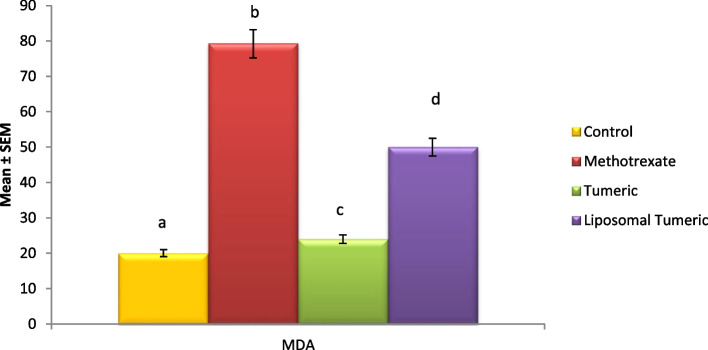
Impact of turmeric and liposomal turmeric on serum malondialdehyde level post methotrexate intoxication. Data are expressed as mean ± SEM (*n* = 10). *p* ≤ 0.05 value is considered significant. Groups having the same letter are not significantly different from each other, while those having different letters are significantly different from each other

**Fig. 2:**
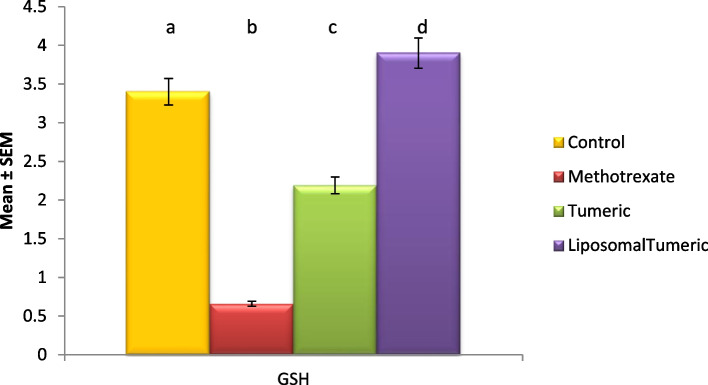
Impact of turmeric and liposomal turmeric on serum reduced glutathione activity post methotrexate intoxication. Data are expressed as mean ± SEM (*n* = 10). *p* ≤ 0.05 value is considered significant. Groups having the same letter are not significantly different from each other, while those having different letters are significantly different from each other

Methotrexate intoxication induced a significant elevation in oxidative stress biomarker including MDA, and a significant reduction in antioxidant biomarker reduced glutathione with a mean value of 79.2 and 0.66, respectively, as compared to the control value. Meanwhile, a concomitant modulation in these biomarkers was observed post turmeric treatment with a mean value of 24 and 2.19, respectively, and liposomal turmeric treatment with a mean value of 50 and 3.9, respectively, as compared with the MX-intoxicated group, with the liposomal turmeric showing the most significant effect implying its antioxidant power as shown in Figs. 1 and 2.

### Modulation of autophagy biomarkers

**Fig. 3:**
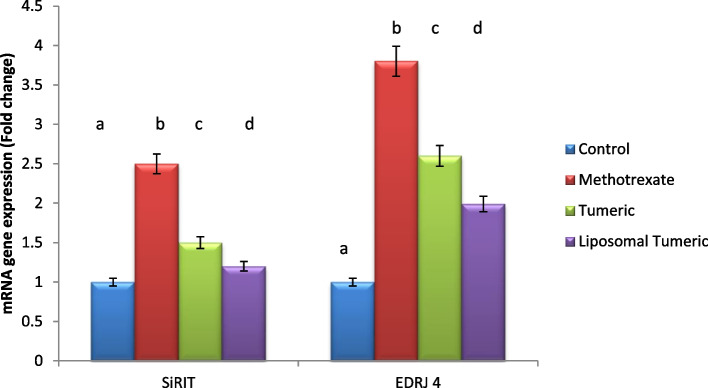
Impact of turmeric and liposomal turmeric on serum autophagy markers SiRT-1 and ERDJ-4 gene expression post methotrexate intoxication. GAPDH was used as an internal control for calculating mRNA fold changes. Data are expressed as mean ± SEM (*n* = 10). *p* ≤ 0.05 value is considered significant. Groups having the same letter are not significantly different from each other, while those having different letters are significantly different from each other

Methotrexate intoxication deduced a significant elevation in autophagy biomarkers including SiRT-1 and ERDJ-4 gene expression by 2- and 4-fold, respectively, as compared to the control value. Meanwhile, a concomitant reduction in these biomarkers was observed post turmeric treatment by a value of 1-fold for both and liposomal turmeric treatment by a value of 2-fold for both, respectively, as compared with the MX-intoxicated group, with the liposomal turmeric showing the most significant effect reflecting the role of autophagy in turmeric therapy as revealed in (Fig. 3).

### Modulation of apoptosis

**Fig. 4:**
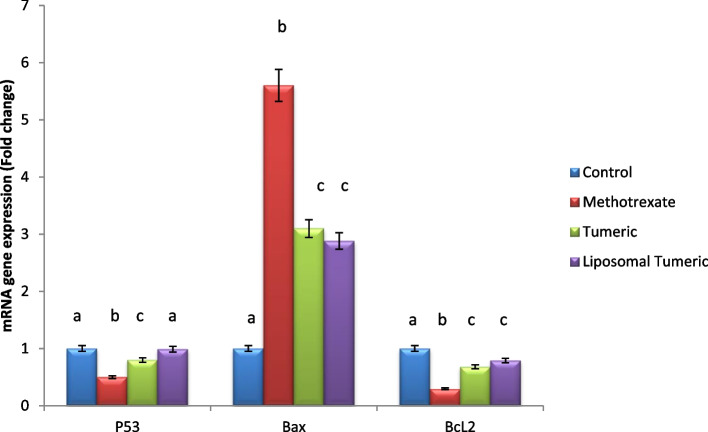
Impact of turmeric and liposomal turmeric on serum apoptotic markers P53, Bax, and BcL2 gene expression post methotrexate intoxication. GAPDH was used as an internal control for calculating mRNA fold changes. Data are expressed as mean ± SEM (*n* = 10). *p* ≤ 0.05 value is considered significant. Groups having the same letter are not significantly different from each other, while those having different letters are significantly different from each other

Methotrexate intoxication demonstrated a significant elevation in the gene expression of apoptotic biomarker, namely Bax, and a significant reduction in antiapoptotic marker Bcl2 and P53 by 6-, 3-, and 5-fold, respectively, as compared to the control value. Meanwhile, a concomitant modulation in these biomarkers was observed post turmeric treatment via 3-, 2-, and 3-fold, respectively, and liposomal turmeric treatment by 3-, 5-, and 4-fold, respectively, as compared with the MX-intoxicated group, with the liposomal turmeric showing the most significant effect deducing its antiapoptotic effect as represented in Fig. 4.

### Modulation of inflammation

**Fig. 5:**
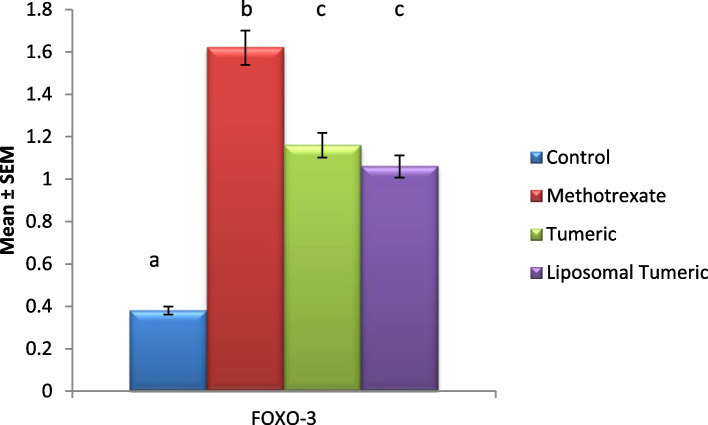
Impact of turmeric and liposomal turmeric on serum inflammatory marker FOXO-3 protein expression post methotrexate intoxication. Data are expressed as mean ± SEM (*n* = 10). *p* value is considered significant. Groups having the same letter are not significantly different from each other, while those having different letters are significantly different from each other

Methotrexate intoxication elucidated a significant elevation in the protein expression of the inflammatory marker FOXO-3 by a mean value of 1.62 as compared to the control value. Meanwhile, a concomitant modulation in this inflammatory marker was observed post turmeric and liposomal turmeric by a mean value of 1.16 and 1.06, respectively, as compared with the MX-intoxicated group, with the liposomal turmeric showing the most significant effect in this aspect reflecting the anti-inflammatory effect of turmeric as highlighted in Fig. 5.

### Modulation of BNP

**Fig. 6:**
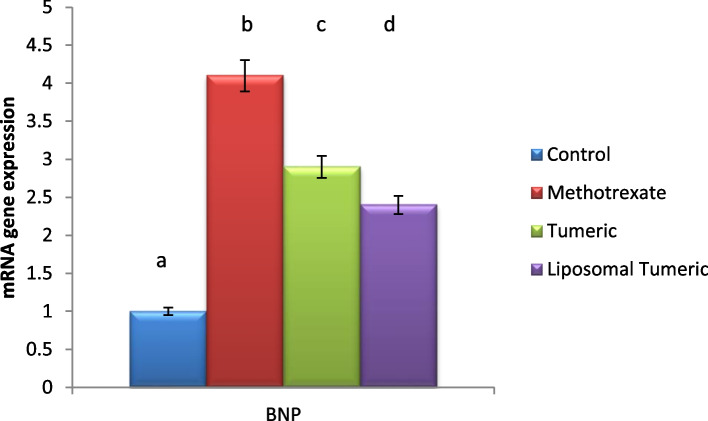
Impact of turmeric and liposomal turmeric on serum brain natriuretic peptide (BNP) gene expression post methotrexate intoxication. GAPDH was used as an internal control for calculating mRNA fold changes. Data are expressed as mean ± SEM (*n* = 10). *p* ≤ 0.05 value is considered significant. Groups having the same letter are not significantly different from each other, while those having different letters are significantly different from each other

Methotrexate intoxication elucidated a significant elevation in the gene expression of the brain biomarker BNP by a value of 4-fold as compared to the control value. Meanwhile, a concomitant modulation in this biomarker was observed post turmeric and liposomal turmeric by a value of 1- and 2-fold, respectively, as compared with the MX-intoxicated group, with the liposomal turmeric treatment showing the most significant impact as shown in Fig. 6.

## Discussion

MX, a competitive inhibitor of dihydrofolate reductase, can prevent the production of DNA, RNA, thymidylate, and proteins. For this reason, MX is generally utilized in cancer therapy besides nonneoplastic conditions, including psoriasis and rheumatoid arthritis [2]. Previous evidences elucidated that toxicity of the lung, liver, brain, and kidney is the main limit for MX application [1]. To understand MX toxicity, a number of mechanisms have been inspected. One of them is disturbing the antioxidant defense mechanism, contributing to oxidative stress-induced damage in several organs and crosslinked autophagy [14].

The current study revealed that MX intoxication deduced a significant elevation in AUTP biomarkers including SiRT-1 and ERDJ-4. Meanwhile, a concomitant reduction in these biomarkers was observed post turmeric and liposomal turmeric treatment; this coincides with the hypothesis that MX induced reduction in the antioxidant defense mechanism contributing to augmenting the release of ROS that subsequently promotes mitochondrial malfunction and endoplasmic reticulum stress accompanied with exaggerated production of ROS resulting in hepatotoxicity, parenchymal lung injury, and interstitial and alveolar fibrosis [15, 16]. It was evidenced that mitochondrial malfunction and simultaneous ROS production can arbitrate autophagic cell death [4]. AUTP is an extremely conserved mechanism that maintains energy resources and ruins unnecessary products. It is suitable for relieving stressful conditions as oxidative stress and ER stress. m-TOR is the mammalian target of rapamycin and considered as one of the detectors regulating autophagy. SiRT-1/AKT/mTOR pathway is implied in numerous fibrotic conditions, as liver, cardiac, kidney, and pulmonary fibrosis [17]. Grounded on former findings, it is worthy to investigate the efficacy of MX on AUTP amelioration via exploring SiRT pathway and its consequence on ERDJ expression that is contributed in toxic influence in addition to inspecting the potential effect of liposomal turmeric in ameliorating these injurious effects. Rationale on this study relies on bioinformatics examinations to reclaim a set of distinctive autophagy genes SiRT-1, ERDJ-4, P53, and FOXO-3. Furthermore, to authorize the efficacy of liposomal turmeric on modifying the expression of the selected autophagy genes, ERDJ-4, SiRT expression, and oxidative stress are induced in rat model. Moreover, the effect of MX, turmeric, and liposomal turmeric on the apoptotic features including P53, Bax, and BCL2 was evaluated. Turmeric, a naturally existing coenzyme, is an effective and safe remedial antioxidant by being a free radical scavenger which inhibits oxidative injuries of lipids, DNA, proteins, and other necessary molecules through promoting Nrf2-related genes. Turmeric can inhibit the development of lung and liver fibrosis via its antioxidant and anti-inflammatory properties through reducing various inflammatory mediators as FOXO-3, TGF-β, TNF-α, and MCP1 in the lung and liver [7, 18].

Hsp70 protein action is adjusted via accessory proteins, contributing members of the DnaJ-like protein family. Characterized by the presence of a highly conserved 70-amino acid J domain, DnaJ homologs promote Hsp70 ATPase activity to further steady their collaboration with unfolded substrates [19]. DnaJ homologs are contributed in almost all facets of protein folding and synthesis and exist in vast organelles. Inside the endoplasmic reticulum, DnaJ homologs can assist in the secretion, translocation, retro-translocation, and ER-associated degradation of secretory pathway proteins. ERdj4 was extremely promoted at both the protein and mRNA level as a result of ER stress, designating that DnaJ may be tangled in protein folding or ER-associated degradation [20].

The present study elucidated that methotrexate intoxication elucidated a significant elevation in apoptotic biomarkers including Bax, P53, and a significant reduction in antiapoptotic marker Bcl2. Meanwhile, a concomitant modulation in these biomarkers was observed post turmeric and liposomal turmeric treatment; this coincides with the thesis that the suppressor gene p53 is mutated in nearly 50% of human cancers and promotes autophagy. It was estimated that P53 purposes as an essential arbitrator for damage-promoted apoptosis and has been shown to induce autophagy in damage-regulated autophagy modulator (DRAM)-dependent manner to elicit apoptosis in human cancer cell lines [21]. DRAM is a lysosomal integral membrane protein and a direct target of p53-induced macroautophagy and helps in accumulation of autophagosomes [22]. The p53-mediated apoptosis involves several chromatin-remodeling factors [14, 23] (e.g., e2f1 as it involves in transcriptional repression of cell proliferation as part of component with retinoblastoma complex). Autophagy genes are organized by several factors contributing to chromatin remodeling as p53 functions at upstream of autophagy signaling pathway to promote apoptosis [4].

Herein, methotrexate intoxication illustrated a significant elevation in inflammatory biomarker FOXO-1. Meanwhile, a concomitant modulation in this inflammatory marker was observed post turmeric and liposomal turmeric treatment; this is in parallel with the hypothesis that FOXO and ROS can control AUTP. Lately, it has been hypothesized that transcriptional level of several autophagy-related genes was elevated via activated FOXO [24]. The genes included were EDRJ-4, Atg8/LC3, Atg12, Vps34, SiRT-1, and Atg6 to induce protein degradation in atrophying muscle cells, and surprisingly, the regulatory role of FOXO on Atg8 and Atg12 seems to be direct [25]. The chromatin-remodeling factors deacetylate FOXO transcription factors and promote longevity in worms, flies, and mammals [26, 27]. Recent evidence illustrated that in mice, sirtuin-type Sirt1 is able to induce autophagy in both normal growth and starvation conditions. SiRT-1 acetylates Atg5, Atg7, and Atg8 autophagy factors in an NADP-dependent manner [28]. The depletion in Sirt1 function in mutant phenotypes resembles those of autophagy-defective mice, such as early death and the accretion of dysfunctional mitochondria. Methotrexate intoxication elucidated a significant elevation in oxidative stress biomarker malondialdehyde and a consignment reduction in antioxidant biomarker GSH. Meanwhile, a concomitant modulation in this oxidative stress biomarker was observed post turmeric and liposomal turmeric treatment with liposomal turmeric showing the most significant effect; this is reflected with the study that ROS can regulate starvation-induced autophagy. Recent evidence shows that Atg4 and SiRIT, essential proteases in the autophagy machinery, have been recognized as a direct target for oxidation by ROS. Accumulation of ROS released throughout numerous cellular activities mostly by respiration contributes to oxidative stress. Cells respond to oxidative stress by activating numerous defense mechanisms. Previous studies deduced that ROS behave as signaling molecules and can induce autophagy contributing to subsequent loss of the affected cells. Autophagy has a precarious function in the cellular interaction to oxidative stress [29].

Mammalian cells utilize specified and complicated machinery for discarding disformed proteins or deformed organelles. Such machinery is a portion of a mechanism named autophagy [30]. Furthermore, if autophagy is precisely functioned to remove dysfunctional mitochondria, it is here named mitophagy. Autophagy and mitophagy have essential physiological implications involved in cellular differentiation, resistance to stresses such as metabolic control, starvation, and adaptation to the varying environmental conditions. Regrettably, converted cancer cells frequently exploit autophagy and mitophagy for sustaining their metabolic reprogramming and growth to a point that autophagy and mitophagy are recognized as promising targets for ongoing future antitumoral therapies [31]. Sirtuins are NAD+-dependent deacylases with a fundamental role in sensing and modulating cellular response to external stresses such as nutrients availability and therefore involved in oxidative stress control, aging, inflammation, cancer, and differentiation [32]. It is obvious, therefore, that autophagy, mitophagy, and sirtuins share many common aspects to a point that, recently, sirtuins have been linked to the control of mitophagy and autophagy. In the context of cancer, such an impact is attained by modulating transcription of mitophagy and autophagy genes, by posttranslational modification of proteins belonging to the mitophagy and autophagy machinery, and by adjusting ROS formation or major metabolic pathways such as glutamine metabolism or Krebs cycle. The role of sirtuins, autophagy, and mitophagy in cancer and how sirtuins can regulate autophagy and mitophagy in cancer cells were illustrated. Lastly, sirtuin’s role in the context of tumor progression and metastasis signifies glutamine metabolism as a model of how a concerted promoting or impairing sirtuins in cancer cells can control mitophagy and autophagy by interrupting the metabolism of this essential amino acid [33].

Sirtuins are considered as class 3 histone deacetylases; their enzymatic activity relies on cofactor NAD^+^ [34]. Sirtuins were stated to control various activities via regulating DNA repair, metabolism, gene expression, oxidative stress response, mitochondrial function, and biogenesis. Disregulation of sirtuins action and expression may contribute to tissue-specific degenerative events involved in the development of several human pathologies, including neurodegeneration, cancer, and cardiovascular disease. Sirtuin 1 is the furthermost studied member of this class of enzymes, whose expression is concomitant with increased insulin sensitivity. SiRT-1 has been implicated in both anticancer and tumorigenic processes and is further stated to control essential metabolic pathways. Via regulating p53 deacetylation and modulation of autophagy, SiRT-1 is involved in lifespan extension and cellular response to caloric restriction. Lately, scientific interest focusing on the identification of SiRT-1 modulators has led to the discovery of novel small molecules targeting SiRT-1 activity [23]. Former studies reported the effect of nutraceuticals in upregulating SiRT-1 activity, including polyphenolic products in vegetables, plants, and fruits, including turmeric, resveratrol, and quercetin [35].

The utmost considered member of this enzymatic class is SiRT-1. SiRT-1 regulates inflammation, metabolic pathways, cell survival, and cellular senescence and participate in the pathogenesis of chronic conditions such as diabetes as well as neurodegenerative, pulmonary, and cardiovascular diseases. Indeed, SiRT-1 has been reported to play a key role in tumorigenesis, as an oncogene or tumor suppressor [35]. SiRT-1 can adjust these processes via deacetylation of lysine groups of non-histone in addition to histone proteins, comprising recognized transcription factors (FOXO, p53, MyoD, PGC-1α) [36].

Oxidative stress plays a vital part in the pathogenesis of most cases. Excess ROS, RNS, and free radicals contribute to destroying cellular contents, including DNA, proteins, and lipids. Imbalance between antioxidants and oxidants contributes into cellular dysfunction, necrosis, and apoptosis [37].

SiRT-1 controls SOD and GPX expression [38]. Moreover, since mitochondrial dysfunction contributes to enhancing apoptosis, SiRT-1 control apoptosis via modifying PGC-1α acetylation [39]. SiRT-1 as well controls inflammatory response [40]. By modifying NF-κB and p53 acetylation, SiRT1 control transcription of genes such as FOXO-3, TNF-α, IL-8, IL-6, and IL-1 [41–43] via NF-κB and SiRT-1 also regulates the expression of genes such as promotor of apoptosis Bax and inhibitor of apoptosis protein (IAP), Bcl-2, and TNF receptor [44].

SiRT-1 fight against oxidative stress via modulating the acetylation of FOXO, as it participates in apoptosis, antioxidant progressions and cell proliferation [45]. Through promoting FOXO/MsSOD pathway, SiRT-1 promote catalase and MnSOD expression, by deacetylating p53, thus enhancing cellular antioxidant capacity [44, 46, 47], countering oxidative stress, and enhancing damage repair [48].

Along the preceding few eras, the eternally growing consciousness is that bioactive diet components can behave as anti-inflammatory, apoptotic, and antioxidant negotiators, thereby dropping the deleterious properties of oxidative tension and the prevalence of chronic maladies, for instance, cancer, diabetes, and hepatic and cardiovascular complaints [49]. Quite a lot of nutraceuticals can moderate SiRT-1 action [50, 51]. For instance, turmeric is considered as a natural polyphenol [52]. As an antioxidant, turmeric is able to eliminate ROS and RNS by increasing the expression of antioxidant proteins via induction of upstream coding genes such as nuclear factor erythroid 2-related factor 2 (Nrf2), Kelch-like ECH-associated protein 1, and antioxidant response element [7] that promote the transcription of antioxidant genes such as SOD, GPx, GST, and GSH and of phase 2 detoxifying enzymes such as HO-1, NADPH, and NAD(P) H dehydrogenase (quinone)-1 [53].

Turmeric possesses immunomodulatory effect via intermingling with factors tangled in inflammatory interaction as STAT/JAK pathway, inhibitor of cytokine signaling (SOCS) expression, and FOXO-3/NF-κB/TLR-4/MyD-88 axis. Turmeric inhibits phosphorylation of STAT/JAK by binding with its α,β-unsaturated carbonyl portion to residue 259 of cysteine in STAT3 with consequent activation. In vitro turmeric can phosphorylate STAT3 and mitigate inflammatory interaction [54]. In vivo turmeric renovates immunological equilibrium via working on STAT-3/JAK/SOCS signaling pathway, inhibiting STAT3, JAK2, and STAT6 phosphorylation, and elevating PIAS3, SOCS1, and SOCS3 gene expression [55]. Turmeric is able to act on apoptosis and on mitochondrial biogenesis and dysfunction through SiRT activation by small molecules [56]. Turmeric reduces transcription factors, TNF, growth factor receptors, and NOS expression and elevates AMPK levels. In vascular smooth muscle cells, turmeric stimulates AMPK activation that subsequently promotes ATP and superoxide release elevating NAD^+^ levels and SiRT1 activation [8]. Turmeric-induced SiRT-1 upregulation has advantageous influence versus a wide range of maladies counting diabetes, cardiac fibrosis, and hepatic and ischemia/reperfusion injury [8, 57]. Cardiomyocytes cured via turmeric displayed upregulation in SiRT-1, FOXO-3, SDH, BcL2, and COX-2 levels and downregulation in apoptotic markers P53 and Bax. These signs were eradicated once cardiomyocytes were cured via SiRT-1 siRNA [57].

Turmeric is a SiRT modulator natural product that exhibits poor solubility, and bioavailability utilizing higher bioavailable liposomal loaded preparations of turmeric derivatives may result in elevating SiRT-1-activating action, further authenticating the link between SiRT-1, turmeric, and therapeutic effects [57]. The fact that studies indicate that the widely held SiRT activators exert both direct activating effects and indirect effects via modulation of SiRT1 downstream pathways complicates the interpretation of results and, particularly, the mining of data specifically dependent on direct SiRT-1 binding and activation [8].

## Conclusion

Liposomal loaded turmeric may be a promising candidate for preventing methotrexate intoxication via interfering with apoptosis, oxidative stress, autophagy, and inflammatory signaling pathways. Further studies may be required and further investigations.

## Data Availability

No additional data or information is available for this paper.

## Abbreviations

MX: Methotrexate
FOXO: Forkhead box transcription factors
Bax: BCL2-associated X protein
Bcl2: B-cell lymphoma 2
BNP: Brain natriuretic peptide B
Sirt-1: Sirtuin silent mating-type information regulation 2 homolog 1
ERDJ: Endoplasmic reticulum localized DnaJ homologs
P 53: Tumor protein P53
AUTP: Autophagy
CMA: Chaperone-mediated autophagy
Atg5: Autophagy related 5
ROS: Reactive oxygen species
NFκB: Nuclear factor kappa-B
TNF-α: Tumor necrosis factor-α
STAT-3: Signal transducer and activator of transcription
JAK-2: Janus kinase 2
ARE: Antioxidant response element
